# The Aminoalkylindole BML-190 Negatively Regulates Chitosan Synthesis via the Cyclic AMP/Protein Kinase A1 Pathway in Cryptococcus neoformans

**DOI:** 10.1128/mBio.02264-19

**Published:** 2019-12-17

**Authors:** Brian T. Maybruck, Woei C. Lam, Charles A. Specht, Ma. Xenia G. Ilagan, Maureen J. Donlin, Jennifer K. Lodge

**Affiliations:** a Department of Molecular Microbiology, Washington University School of Medicine, St. Louis, Missouri, USA; b Department of Medicine, University of Massachusetts Medical School, Worcester, Massachusetts, USA; c Department of Biochemistry and Molecular Biophysics, Washington University School of Medicine, St. Louis, Missouri, USA; d Department of Biochemistry and Molecular Biology, Saint Louis University, St. Louis, Missouri, USA; Duke University Medical Center

**Keywords:** *Cryptococcus neoformans*, cAMP, chitosan, drug screening, flow cytometry

## Abstract

Cryptococcus neoformans can cause fatal meningoencephalitis in patients with AIDS or other immunocompromising conditions. Current antifungals are suboptimal to treat this disease; therefore, novel targets and new therapies are needed. Previously, we have shown that chitosan is a critical component of the cryptococcal cell wall and is required for survival in the mammalian host and that chitosan deficiency results in rapid clearance from the mammalian host. We had also identified several specific proteins that were required for chitosan biosynthesis, and we hypothesize that screening for compounds that inhibit chitosan biosynthesis would identify additional genes/proteins that influence chitosan biosynthesis. To identify these compounds, we developed a robust and novel cell-based flow cytometry screening method to identify small-molecule inhibitors of chitosan production. We screened the ICCB Known Bioactives library and identified 8 compounds that reduced chitosan in C. neoformans. We used flow cytometry-based counterscreens and confirmatory screens, followed by a biochemical secondary screen to refine our primary screening hits to 2 confirmed hits. One of the confirmed hits that reduced chitosan content was the aminoalkylindole BML-190, a known inverse agonist of mammalian cannabinoid receptors. We demonstrated that BML-190 likely targets the C. neoformans G-protein-coupled receptor Gpr4 and, via the cyclic AMP (cAMP)/protein kinase A (PKA) signaling pathway, contributes to an intracellular accumulation of cAMP that results in decreased chitosan. Our discovery suggests that this approach could be used to identify additional compounds and pathways that reduce chitosan biosynthesis and could lead to potential novel therapeutics against C. neoformans.

## Abstract (precis)

Cryptococcus neoformans is a fungal pathogen that kills ∼200,000 people every year. The cell wall is an essential organelle that protects fungi from the environment. Chitosan, the deacetylated form of chitin, has been shown to be an essential component of the cryptococcal cell wall during infection of a mammalian host. In this study, we screened a set of 480 compounds, which are known to have defined biological activities, for activity that reduced chitosan production in C. neoformans. Two of these compounds were confirmed using an alternative method of measuring chitosan, and one of these was demonstrated to impact the cAMP signal transduction pathway. This work demonstrates that the cAMP pathway regulates chitosan biosynthesis in C. neoformans and validates that this screening approach could be used to find potential antifungal agents.

## INTRODUCTION

Despite the current antifungal therapies, up to 19% of AIDS-related deaths (∼200,000) occur globally due to cryptococcal meningitis (1–3). Several factors contribute to this high mortality rate, including (i) toxicities from current therapies such that many patients cannot tolerate the regimen (2, 3), (ii) the relative expense and difficulty of administering some of the formulations, and (iii) the long duration of the regimen reducing patient compliance. Although the implementation of antiretroviral therapy (ART) has been profound in its success in reducing AIDS-related deaths (1), to avoid immune reconstitution inflammatory syndrome (IRIS) that is linked to ART, ART must be delayed until successful antifungal therapy can be applied (4, 5). With the suboptimal efficacy of current antifungal therapies, it can be challenging to avoid the effects of IRIS. Furthermore, antimicrobial drug resistance is an identified concern when using the current antifungal therapies against cryptococcosis (6, 7). Overall, these concerns create the urgency for the discovery of novel targets for the development of effective drugs against the primary etiology of AIDS-related cryptococcal meningitis, Cryptococcus neoformans.

Chitosan is a key component of the C. neoformans cell wall and is essential for fungal survival in the mammalian host (8, 9). The precursor to chitosan in C. neoformans is chitin. Chitin is synthesized by one or more of the integral membrane proteins, chitin synthases (Chs), some of which require an accessory protein (Csr), from cytoplasmic stores of UDP *N*-acetylglucosamine and is then deacetylated by chitin deacetylases (Cda) to produce chitosan. Although most fungi have genes encoding putative chitin deacetylases, chitosan has been shown to be present in vegetatively growing cells of a small subset of fungi, and chitosan biosynthesis has been studied for only a few species. Chitin is made up of beta-(1,4)-linked *N*-acetylglucosamine (GlcNAc) monomers, and when greater than 50% of these monomers are deacetylated by chitin deacetylases to glucosamine (GlcN [8, 9]), the polymer becomes chitosan. *Cryptococcus* has eight chitin synthase genes, and only one of them (*CHS3*) affects chitosan production (10). Deletion of the *CSR2* gene, which encodes the accessory protein suggested to be associated with Chs3, also prevents chitosan production. The chitin produced by Chs3/Csr2 is subsequently deacetylated into chitosan by three chitin deacetylases: Cda1, Cda2, and Cda3 (9, 10).

Three C. neoformans strains have been engineered to be deficient in chitosan production, via gene deletions of *CHS3*, *CSR2*, or all three *CDA* genes. Chitosan in these three strains was undetectable or very low by biochemical assay or using a chitosan-specific stain (9, 10). The phenotypes of these strains demonstrated that chitosan is important for (i) cell wall integrity, (ii) cell budding, (iii) cell size and morphology, and (iv) virulence (8, 9). Previously we demonstrated that in a mouse inhalation model of infection, chitosan-deficient strains are avirulent and can be cleared within 2 days (8). We examined the host immune response to infection with the *cda1*Δ *cda2*Δ *cda3*Δ (*cda1Δ2Δ3Δ*) chitosan-deficient strain and found an increase in the Th1-mediated immune response. Mice infected with this strain were also fully protected from subsequent challenge with wild-type KN99α (11). We, along with other groups, have shown that these immunological responses are necessary for protection from subsequent C. neoformans infections (12, 13). Taken together, these results identify chitosan as being important for C. neoformans virulence.

Based on the rapid clearance of the *cda1*Δ *cda2*Δ *cda3*Δ chitosan-deficient strain, we hypothesize that compounds that interfere with chitosan biosynthesis and cause chitosan deficiency could facilitate clearance of C. neoformans and that these compounds would provide clues to additional pathways and proteins that regulate or contribute to chitosan production. Therefore, we developed a novel, medium-throughput, cell-based, phenotypic flow cytometry screening assay to identify small molecules that reduce chitosan levels. We screened the Institute of Chemistry and Cell Biology (ICCB) Known Bioactives library and discovered that the aminoalkylindole BML-190 targets the C. neoformans G-protein-coupled receptor (GPCR) Gpr4 and, via the cyclic AMP (cAMP)/protein kinase A (PKA) signaling pathway, contributes to an intracellular accumulation of cAMP that results in decreased chitosan.

## RESULTS

### A novel medium-throughput flow cytometry assay that robustly measures chitosan levels by BR fluorescence.

To identify small molecules that can inhibit chitosan production, we developed a cell-based phenotypic medium-throughput flow cytometry screen that was identified as robust, meaning that it has high precision and good dynamic range, based upon calculating the Z-prime, a calculation commonly used to assess the quality of higher-throughput screening assays (14). Assays with Z-primes greater than 0.5 are considered robust. The chitosan-selective fluorescent dye used for this assay was Cibacron brilliant red (BR) (see Fig. S1A in the supplemental material). BR is an anionic dye that binds selectively to chitosan and has been used previously in chitosan assays (15, 16). Staining occurs by an ionic bond forming between the anionic dye and the protonated amino groups from chitosan.

10.1128/mBio.02264-19.1 FIG S1 Known differential expression of chitosan by C. neoformans strains was identified by flow cytometry. Flow cytometry-generated BR fold fluorescence (A) shows a correlation with the MBTH Chitosan assay (B) when identical strains of C. neoformans are used for both assays. Means among groups were compared to that of the KN99α group using a one-way ANOVA followed by the Bonferroni multiple-comparison test. *, *P* < 0.05; **, *P*< 0.01; ***, *P* < 0.001; ***, *P* < 0.0001. (C) Based upon a Pearson’s *r* correlation analysis, chitosan measured by flow cytometry and the MBTH strongly and statistically covary with one another (*r* = 0.8789; *n* = 12; critical *r* = 0.576; *P* < 0.001). Both conditions are grown in complete RPMI for 3 days at 37°C and 5% CO_2_. BR fold fluorescence = geometric mean fluorescence intensity (gMFI) of BR-stained strain/gMFI of unstained KN99α strain. Download FIG S1, JPG file, 0.04 MB. Copyright © 2019 Maybruck et al. 2019 Maybruck et al. This content is distributed under the terms of the Creative Commons Attribution 4.0 International license.

We confirmed that this flow cytometry-based assay reliably detects differences in chitosan contents of strains of *Cryptococcus* by comparing it to the low-throughput 3-methyl-2-benzothiazolone hydrazone hydrochloride (MBTH) chitosan assay (17, 18). We found a statistically significant positive correlation between the fluorescence from BR-stained C. neoformans strains that differentially produce chitosan, measured via flow cytometry, compared to the levels of chitosan in the same strains, as measured by the MBTH assay (Fig. S1B and C). The key advantages of the flow cytometry-based assay over the MBTH assay are its miniaturized, microplate format (96 well), increased throughput, and the ability to measure multiple parameters from a single sample/well.

### Screening strategy to identify small-molecule inhibitors of chitosan synthesis in C. neoformans.

We chose to screen the 480 compound Institute of Chemistry and Cell Biology (ICCB) Known Bioactives library because the biological targets of the compounds in this library are known, allowing us to potentially identify the mechanism of action of our hits. We developed a screening strategy funnel (Fig. S2) that uses a series of assays that are increasingly sensitive in the ability to identify small-molecule inhibitors of chitosan production so primary hits from the flow cytometry assay could be confirmed by orthogonal approaches.

10.1128/mBio.02264-19.2 FIG S2 Screening strategy funnel for inhibitors of chitosan production from C. neoformans. Shown is an overview of the the primary cell-based phenotypic screen of the 480 compounds from the ICCB library. From our primary screen, we identified 8 hits. Of those 8 hits, 6 were further validated to identify 2 hits. Those hits were identified as confirmed hits by our MBTH chitosan assay: dipyridamole and BML-190. Download FIG S2, JPG file, 0.03 MB. Copyright © 2019 Maybruck et al. 2019 Maybruck et al. This content is distributed under the terms of the Creative Commons Attribution 4.0 International license.

There are five proteins that are already known to affect chitosan production: Chs3, Csr2, Cda1, Cda2, and Cda3 (8–10). Ideally, the screen would identify inhibitors of each of these proteins, along with other, as yet unknown, proteins or pathways. However, the Cdas are functionally redundant, making it less likely that the screen would identify a deacetylase inhibitor. Therefore, we did our initial screening with a strain that expressed only the *CDA1* gene. We chose *CDA1* because it is upregulated in the mouse upon infection and deletion of *CDA1* results in avirulence, while deletion of both *CDA2* and *CDA3* maintains virulence similar to that of the wild type (18). Therefore, for screening, we chose a strain that expresses *CDA1* but has both *CDA2* and *CDA3* deleted (i.e., *cda2Δ3Δ*). The *cda2Δ3Δ* strain produces sufficient chitosan (Fig. S1), retains a wild-type morphology for our screens, and has all the remaining elements of the chitosan production machinery intact, so we should still have been able to identify inhibitors of other factors that impact chitosan production from our screens. The negative control for this screen was the untreated *cda2Δ3Δ* strain, and the positive control was the chitosan-deficient *cda1Δ2Δ3Δ* strain.

**FIG 1:**
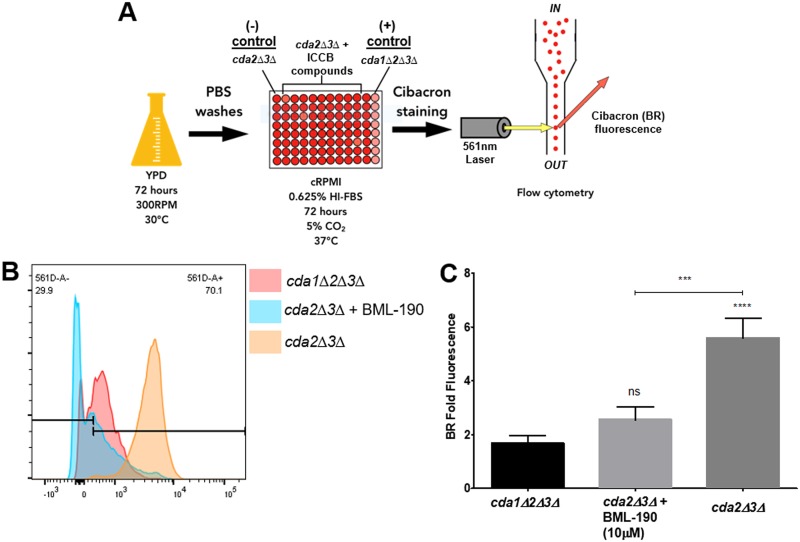
Primary screening strategy. (A) An overview of the primary cell-based phenotypic screen of the 480 compounds from the ICCB library. (B) From each well, 7,500 cell events were collected and further analyzed for geometric mean fluorescence intensity (gMFI) BR fluorescence. (C) Upon comparison to controls, BML-190 was identified as a tentative hit. Cibacron BR fold fluorescence = gMFI of BR-stained *cda2*Δ*3*Δ strain with drug/gMFI of unstained *cda2Δ3Δ* strain. Means were compared to that of the positive control using a one-way ANOVA followed by the Bonferroni multiple-comparison test. A *t* test was used to show a difference between our screening strain with and without drug. ns, not significant.

To minimize concerns associated with the viability of screening strains, prior to subjecting them to the primary screening conditions, they were grown in the optimal growth liquid medium yeast extract-peptone-dextrose (YPD) for 3 days at 30°C under constant shaking (300 rpm). Next, they were washed twice in phosphate-buffered saline (PBS) and then added at 5 × 10^5^ cells/ml to 96-well round-bottom plates containing 100 μl of complete RPMI medium (cRPMI) (0.625% heat-inactivated fetal bovine serum [HI-FBS]) and 1 μl of the ICCB library compound for a 1% dimethyl sulfoxide (DMSO) final concentration with varied compound concentrations (i.e., 1 μM, 10 μM, 5 μg/ml, or 50 μg/ml). The varied compound concentrations were based upon what was provided by the supplier. Furthermore, the ICCB library compounds were screened in duplicate to help reduce the likelihood of false negatives. In the context of growth conditions considered, if compounds do have an effect on altering chitosan levels, for them to even have the potential for preclinical to clinical applications, they would ultimately need to do so under host conditions. Therefore, we chose to characterize their effects under conditions that would most closely simulate those of a mammalian host (i.e., complete RPMI, 5% CO_2_, and 37°C). After 3 days under tissue culture conditions, cells were stained with BR and then fixed in 1% paraformaldehyde (PFA) (Fig. 1A).

Plates were then analyzed for BR fluorescence by flow cytometry. Specifically, from each well, 7,500 cell events were collected from a gated region, within the forward scatter (FSC) versus side scatter (SSC) dot plots. Doublet cell events were not excluded since this is a phenotype of our chitosan-deficient *cda1Δ2Δ3Δ* strain. These populations were then examined for BR fluorescence. The amount of BR fluorescence from the screening strain within each well was calculated by determining the BR geometric mean fluorescence intensity (gMFI) from the samples using unstained *cda2Δ3Δ* strain as a negative control to set the gMFI gate (Fig. 1B). The gMFI was then used to calculate BR fold fluorescence by dividing those numbers by the gMFI of our unstained *cda2Δ3Δ* sample on each plate thereby quantifing the relative levels of chitosan production upon exposure of C. neoformans strains to different compounds (Fig. 1C). This medium-throughput screen (12 plates) was completed in ∼6 h and had an average plate Z-prime of 0.51, providing us confidence that we have an efficient and robust assay (14).

We identified initial hits based upon the following criteria: the compound must induce the *cda2Δ3Δ* strain to have a BR fold fluorescence that is (i) statistically different and (ii) at least ∼2-fold lower in signal than the untreated *cda2Δ3*Δ screening strain (Fig. 1C). This resulted in the identification of eight hits from the ICCB library (Table 1). Based upon the stringency of our hit criteria, this equated to a hit rate of 1.7%, which is below what is commonly seen for the screening of the ICCB library (i.e., 2% to 15% [19–23]).

**TABLE 1:** Primary screen ICCB Known Bioactives library hits

Name	Classification	Known mode of action
BML-190	Bioactive lipid	Cannabinoid CB1/CB2 inverse agonist
dl-PDMP	Lipid biosynthesis	Glucosylceramide synthase inhibitor
Quercetin	Kinase inhibitor	Kinase inhibitor
Dipyridamole	Inhibitor	cGMP phosphodiesterase inhibitor
HA1077	Kinase inhibitor	Inhibitor of Rho-dependent kinases
Juglone	Inhibitor	PIN1 inhibitor
Grayanotoxin III	Ion channel ligand	Sodium channels
RK-682	Inhibitor	VHR phosphatase inhibitor

**FIG 2:**
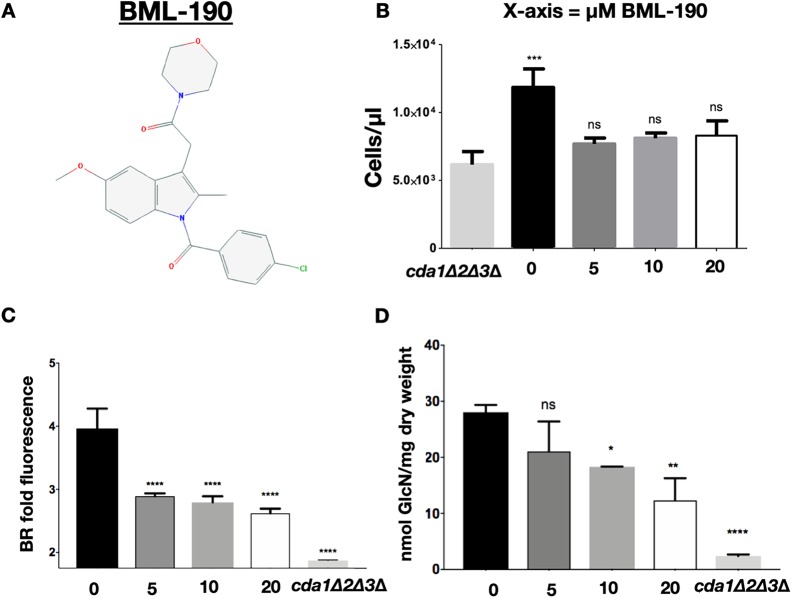
The cannabinoid inverse-agonist BML-190 decreases chitosan levels. (A) Chemical structure of BML-190 (indomethacin morpholinylamide), an aminoalkylindole. (B) C. neoformans concentrations determined by flow cytometry using counting beads with BML-190 concentrations of 0 to 20 μM. (C) Effect of BML-190 on the chitosan levels of wild-type C. neoformans as measured by BR. BR fold fluorescence = gMFI of BR-stained KN99α with BML-190/gMFI of unstained KN99α (*n* = 3). (D) Effects of various doses of BML-190 on the chitosan content as measured by the MBTH assay (*n* = 3). Means among groups were compared to those for the *cda1*Δ*2*Δ*3*Δ strain (B) or KN99α (C and D) with no BML-190 added using a one-way ANOVA followed by the Bonferroni multiple-comparison test.

One effect that the initial hit compounds could have on screening strains is causing their relatively reduced growth or cell death of the *cda2Δ3Δ* strain, unrelated to chitosan deficiency. We anticipated that compounds that recapitulate the phenotype of our chitosan-deficient *cda1Δ2Δ3Δ* strain might have similar, modest growth defects but that the strains should grow as well as or better than the *cda1Δ2Δ3Δ* strain. Compounds that inhibited the growth more than for the *cda1Δ2Δ3Δ* strain likely act through a different mechanism than chitosan deficiency and were eliminated. We also repeated the assay used for the primary screen to generate a dose-response profile for each hit. However, two of the eight compounds, grayanotoxin III and RK-682, were eliminated from this analysis due to their high costs compared to those of the other six hits; these two compounds will be assessed further in the future. Hits were assessed for causing growth inhibition on *cda2∆3∆* or wild-type cells by using flow cytometry to measure the difference in their concentration over 3 days of growth in cRPMI and under tissue culture conditions. Dose response and proliferation validations were done as described above. We validated 2 of the 6 remaining hits (Table 1), BML-190 (Fig. 2) and dipyridamole (Fig. S3).

10.1128/mBio.02264-19.3 FIG S3 The phosphodiesterase inhibitor dipyridamole reduces chitosan in C. neoformans. (A) Chemical structure of dipyridamole. (B) C. neoformans concentrations determined by flow cytometry using counting beads with dipyridamole concentrations between 100 μM and 400 μM that were incubated for 3 days in cRPMI. (C) A dose-response of dipyridamole-cultured C. neoformans (KN99α) incubated for 3 days in cRPMI, stained with BR, and measured for gMFI by flow cytometry. (D) A dose-response of dipyridamole-cultured C. neoformans (KN99α) incubated for 3 days in cRPMI and with chitosan content determined using the MBTH assay. Means among groups (*n* = 3) were compared to values for the *cda1*Δ*2*Δ*3*Δ strain (B) or KN99α (C and D) with no BML-190 added using a one-way ANOVA followed by the Bonferroni multiple-comparison test. Download FIG S3, JPG file, 0.04 MB. Copyright © 2019 Maybruck et al. 2019 Maybruck et al. This content is distributed under the terms of the Creative Commons Attribution 4.0 International license.

### The aminoalkylindole BML-190 reduces chitosan synthesis in C. neoformans.

Our secondary screen used the MBTH biochemical assay to measure chitosan. Therefore, we confirmed the role of BML-190 and dipyridamole in recapitulating the phenotype of our chitosan-deficient strain (i.e., reducing chitosan levels and growth of wild-type C. neoformans, albeit not significantly less than our chitosan-deficient strain) by our secondary screen using the biochemical assay to measure chitosan production rather than the fluorescence detection method that we used for our primary and validating screens (Fig. 2B to D and Fig. S3B to D) and eliminated the four other compounds. BML-190 is known as an inverse agonist of G-protein-coupled receptors (GPCRs) (24). To our knowledge, there was no known connection between GPCRs and chitosan production in C. neoformans; thus, we chose to further characterize the effect of BML-190 in C. neoformans and to explore the potential role of GPCR signaling in the synthesis of chitosan.

### BML-190 reduces chitosan production in multiple research and clinical strains of C. neoformans.

**FIG 3:**
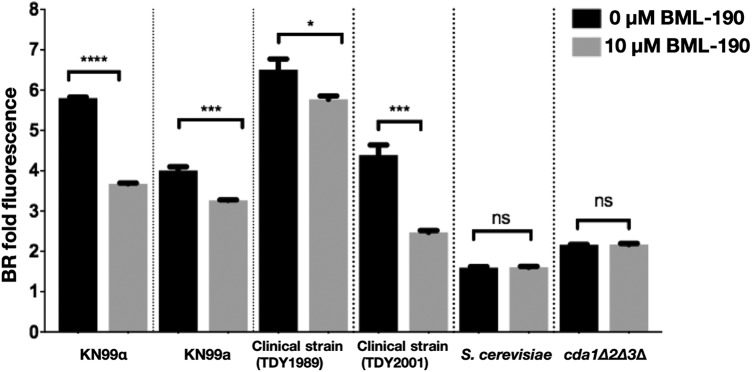
BML-190 causes chitosan reduction in multiple strains. C. neoformans strains were cultured with and without BML-190 (10 μM) for 3 days, stained with BR, and measured for BR fold fluorescence by flow cytometry (*n* = 3). A Student *t* test was used to show a difference between strains with and without drug.

C. neoformans var. *grubii* (serotype A) strains are important contributors to cryptococcosis in humans (25). Two congenic mating strains from this group, KN99α and KN99a, along with clinical strains TDY1989 and TDY2001 were treated with 10 μM BML-190, and all demonstrated a reduction in chitosan production (Fig. 3). However, as expected, no chitosan was detectable with Saccharomyces cerevisiae or our *cda1Δ2Δ3Δ* strain, which are both chitosan deficient. This helps further support the specificity of BR for measuring chitosan since S. cerevisiae is known to produce chitosan only during sporulation (26) and we have empirically shown chitosan deficiency with our *cda1Δ2Δ3Δ* strain.

### BML-190 acts through the G-protein-coupled receptor Gpr4 of C. neoformans.

**FIG 4:**
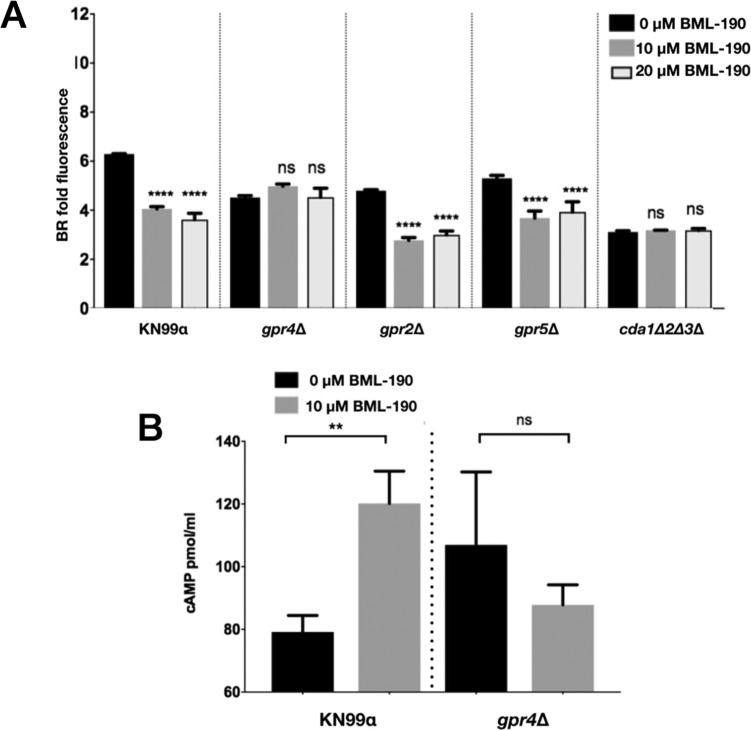
The G-protein-coupled receptor Gpr4 is necessary for BML-190 to reduce chitosan levels. (A) The C. neoformans *gpr*-deficient strains were cultured with and without BML-190 (10 μM and 20 μM) for 3 days, stained with BR, and measured for gMFI by flow cytometry (*n* = 4). (B) BML-190 induces intracellular accumulation of cAMP in wild-type C. neoformans but not in a *gpr4*Δ strain. Means among groups were compared to those of strains with no BML-190 added using a one-way ANOVA followed by the Bonferroni multiple-comparison test or a Student *t* test.

In human cells, BML-190 targets the GPCR cannabinoid receptors 1 and 2 (CNR1/2), with a greater affinity toward CNR2 (24). GPCRs are a family of receptors that are known to be activated by external molecules that are well known to induce signal transduction in the cAMP pathway, which is important for many cellular responses. BML-190 acts as an inverse agonist of this receptor, resulting in increased cAMP levels (24). Since G-protein signaling is known to be conserved among organisms, with particular importance associated with fungal virulence (27, 28), we examined if BML-190’s mode of action could also target GPCRs from C. neoformans (29). Initially, we looked for the closest homologs to CNR1/2 in C. neoformans using BLAST and multiple-sequence alignments. We identified four, GPR2, GPR3, GPR4, and GPR5, although none have more than 30% similarity to the human CNR1/2. To identify the target of BML-190, we used strains that had deletions of each of the four GPCRs. These deletion strains were available from the Madhani UCSF C. neoformans knockout collection obtained from the Fungal Genetics Stock Center (FGSC) (30, 31). After culturing these strains with BML-190 in cRPMI for 3 days under tissue culture conditions, we discovered that of these four, only the *gpr4Δ* strain showed no decrease in chitosan in response to BML-190 (Fig. 4A and Fig. S4). Because the *gpr4Δ* strain was nonresponsive to BML-190, Gpr4 may be the primary receptor for BML-190 in C. neoformans.

10.1128/mBio.02264-19.4 FIG S4 Gpr3 is not a receptor for BML-190. C. neoformans *gpr3Δ* cells were cultured with/without BML-190 (20 μM) for 3 days in cRPMI, stained with BR, and measured for gMFI by flow cytometry. Means among groups (*n* = 6) were compared to those of strains with no BML-190 added using a Student *t* test. Note that this experiment on the *gpr3Δ* strain was done in separate, subsequent experiment and therefore represented in a different figure from the other mutants (Fig. 4). Download FIG S4, JPG file, 0.04 MB. Copyright © 2019 Maybruck et al. 2019 Maybruck et al. This content is distributed under the terms of the Creative Commons Attribution 4.0 International license.

Agonists of cannabinoid receptors are known to contribute to the degradation of intracellular cAMP (32, 33). Similarly, in C. neoformans, Gpr4 activates the cAMP/PKA pathway upon stimulation by the agonists glucose and the amino acid methionine, which ultimately results in cAMP degradation (29, 34). Inversely, if BML-190 targets Gpr4, and acts as an inverse agonist, then it should cause a buildup of cAMP in wild-type cells but not in the *gpr4Δ* strain. Therefore, we determined the intracellular content of cAMP in wild-type C. neoformans and the *gpr4Δ* strain with and without 10 μM BML-190. We incubated strains with and without BML-190 for 30 min, processed cells to stably collect cAMP, and demonstrated that BML-190 causes an increase in intracellular cAMP from wild-type cells but not the *gpr4Δ* strain (Fig. 4B). Based on these results, we hypothesize that BML-190 targets Gpr4 and causes an increase in C. neoformans intracellular cAMP that inversely correlates with a decrease in chitosan.

### BML-190 reduces chitosan by increasing cAMP levels via the cAMP/PKA pathway.

**FIG 5:**
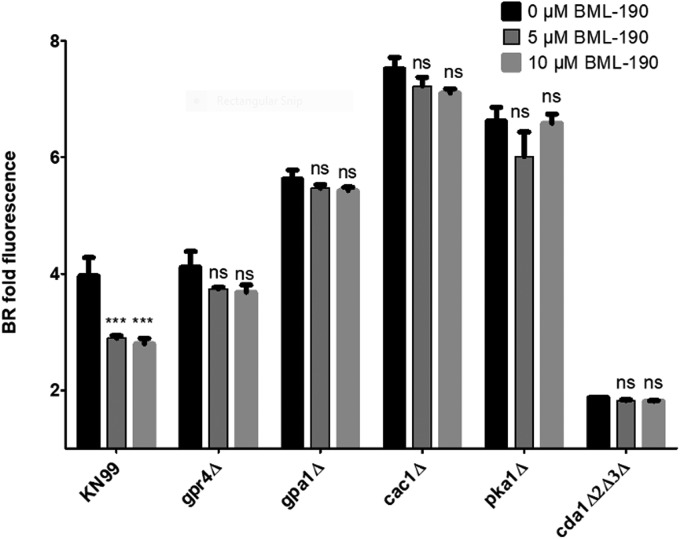
The mode of action for BML-190 to cause chitosan reduction is via the cAMP/PKA pathway. C. neoformans strains deficient in genes from the cAMP/PKA pathway were cultured with and without BML-190 for 3 days in cRPMI (0.625% HI-FBS) at 37°C and 5% CO_2_. These strains were stained with BR and measured for gMFI by flow cytometry. BR fold fluorescence = gMFI of BR-stained strain with BML-190/gMFI of unstained KN99α (*n* = 3). Means among groups were compared to those of strains with no BML-190 added using either a Student *t* test or a one-way ANOVA followed by the Bonferroni multiple-comparison test, where applicable.

**FIG 6:**
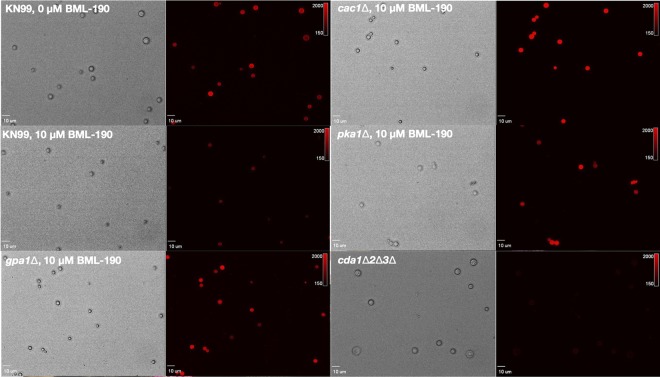
Photomicrographs showing BR fluorescence on some cAMP/PKA pathway mutants with and without BML-190. C. neoformans strains deficient in genes from the cAMP/PKA pathway were cultured with and without 10 μM BML-190 for 3 days in cRPMI (0.625% HI-FBS) at 37°C and 5% CO_2_. These strains were stained with BR and examined by epifluorescence using filter sets for TRITC (excitation, 521 to 565 nm; emission, 553 to 633 nm).

Gpr4 is known to be a receptor in C. neoformans for the cAMP/PKA pathway (29). Therefore, we hypothesized that BML-190 increases cAMP in wild-type *Cryptococcus* by acting as an inverse agonist to the cAMP/PKA pathway, which is responsible for regulating cAMP. To test that idea, we selected three additional strains with deletions of genes in this pathway, including deletions of *GPA1*, *CAC1*, and *PKA1*. Gpa1 is the G-protein alpha subunit of the heterotrimeric G-protein complex that interacts with Gpr4. Upon ligand activation, Gpr4 activates Gpa1 and dissociates it from the complex to continue the signal transduction cascade binding to the adenylyl cyclase (Cac1), which converts ATP to cAMP. Based upon our hypothesis that cAMP levels are inverse to chitosan, the deletion of either *GPA1* or *CAC1* is predicted to decrease cAMP production and increase chitosan. Following the incubation of these different strains for 3 days in cRPMI under tissue culture conditions, the *gpa1Δ* and *cac1Δ* strains both showed an increase in chitosan production compared to that of the wild type, and increasing doses of BML-190 had no effect on chitosan in these deletion strains (Fig. 5). We also confirmed that addition of BML-190 to the *cac1Δ* strain does not affect chitosan levels, as measured by the MBTH biochemical assay (data not shown). Photomicrographs of BR-stained wild-type (KN99α) and mutant strains with and without 10 μM BML-190 further confirmed the results of our flow cytometry analysis (Fig. 6).

Pka1 is the catalytic subunit of protein kinase A, which is activated by the action of cAMP on the regulator subunit Pkr1. Similar to the case with the upstream components of the pathway, the *pka1Δ* strain showed an increase in chitosan, and BML-190 had no effect on chitosan in this strain (Fig. 5), suggesting that Pka1 may be a negative regulator of chitosan production.

Furthermore, through an examination of the other group of G proteins in this signaling pathway, induced by BML-190, chitosan reduction is dependent on the G-protein alpha subunit (Gpa) 1 but not subunit 2 or 3 (Fig. S5). Overall, these results support the idea that BML-190 works through the cAMP/PKA pathway to positively regulate cAMP and to negatively regulate chitosan production.

10.1128/mBio.02264-19.5 FIG S5 BML-190 causes chitosan reduction by working through the G-protein alpha subunit 2 Gpa1. C. neoformans *gpa*-deficient strains were cultured with and without BML-190 (10 μM and 50 μM) for 3 days in cRPMI, stained with BR, and measured for gMFI by flow cytometry. Means among groups (*n* =4) were compared to those of strains with no BML-190 added using a one-way ANOVA followed by the Bonferroni multiple-comparison test. ****, *P* < 0.0001. Download FIG S5, JPG file, 0.04 MB. Copyright © 2019 Maybruck et al. 2019 Maybruck et al. This content is distributed under the terms of the Creative Commons Attribution 4.0 International license.

**FIG 7:**
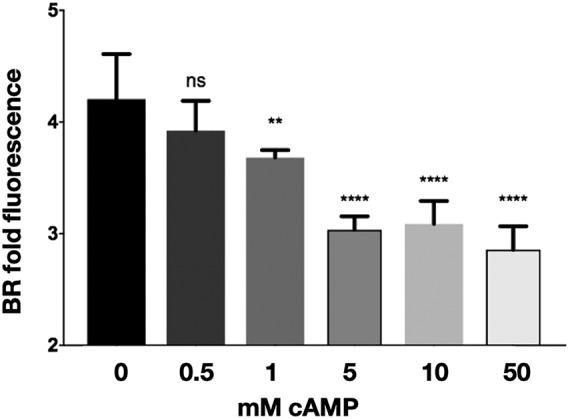
cAMP addition to C. neoformans results in a negative regulation of chitosan. C. neoformans wild-type cells were cultured with increasing concentrations of dibutyryl-cAMP for 3 days, stained with BR, and measured for BR fold fluorescence by flow cytometry. Means among groups (*n* = 5) were compared to those of strains with no cAMP added using a one-way ANOVA followed by the Bonferroni multiple-comparison test.

**FIG 8:**
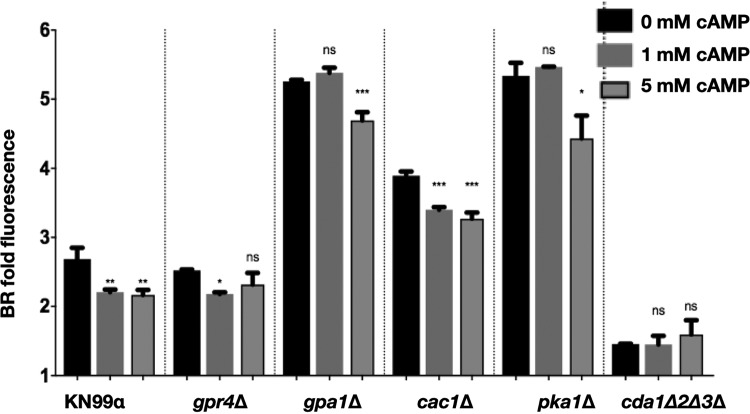
cAMP addition to C. neoformans cAMP/PKA pathway mutants rescues the ability of the pathway to reduce chitosan. C. neoformans cAMP/PKA pathway mutants were cultured with increasing concentrations of dibutyryl-cAMP for 3 days in cRPMI, stained with BR, and measured for BR fold fluorescence by flow cytometry. Means among groups (*n* = 3) were compared to those of strains with no cAMP added using a one-way ANOVA followed by the Bonferroni multiple-comparison test.

If cAMP is important in this pathway for regulating chitosan, we should be able to recapitulate the effects of BML-190 with the addition of cAMP to wild-type C. neoformans. When wild-type cells were incubated with increasing amounts of cAMP for 3 days in cRPMI, under tissue culture conditions, we observed decreasing amounts of chitosan (Fig. 7). This finding is consistent with the hypothesis that BML-190 causes an intracellular accumulation of cAMP resulting in decreased chitosan. We tested this by adding increasing concentrations of cAMP to cAMP/PKA pathway mutants to see if increasing cAMP would reduce chitosan. We saw a modest, but statistically significant, decrease in chitosan in the wild type and the *gpa1*Δ and *cac1*Δ strains at 5 mM dibutyryl-cAMP (Fig. 8). We also saw a smaller, albeit significant, difference in the *pka1*Δ and *gpr4*Δ strains.

The levels of BR fluorescence were higher in the *gpa1*Δ and *cac1*Δ strains, suggesting that the intracellular cAMP levels were lower in these strains than in the wild type or the *gpr4*Δ strain. These data suggest that Cac1 and Gpa1 directly affect cAMP levels. Interestingly, the loss of Gpr4 does not appear to strongly decrease cAMP levels, based on the level of BR fluorescence. It is possible that loss of Gpr4 triggers compensating changes that allow the cAMP/PKA pathway to maintain normal levels of cAMP. Furthermore, since the *pka1*Δ strain is associated with a higher level of chitosan that is attenuated with the addition of cAMP, this implies that the regulation of chitosan is likely to be downstream of Pka1.

**FIG 9:**
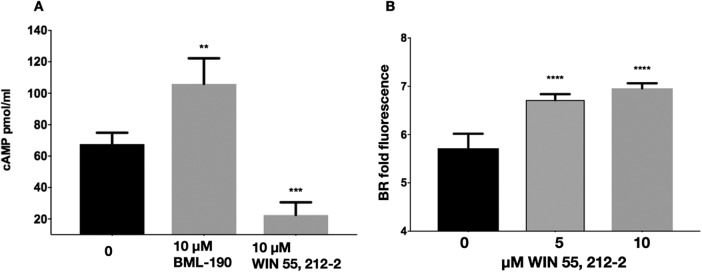
The cannabinoid receptor agonist WIN 55,212-2 causes a decrease in cAMP and subsequent increase in chitosan. (A) Wild-type C. neoformans (KN99α) was cultured for 30 min in cRPMI with either 10 μM BML-190 or WIN 55,212-2 and then collected and lysed for intracellular cAMP competition ELISA (*n* = 5). (B) A dose-response of WIN 55,212-2-cultured C. neoformans (KN99α) incubated for 3 days in cRPMI, stained with BR, and measured for BR fold fluorescence by flow cytometry. Means among groups (*n* = 3) were compared to those of strains with no drug added using a one-way ANOVA followed by the Bonferroni multiple-comparison test.

**FIG 10:**
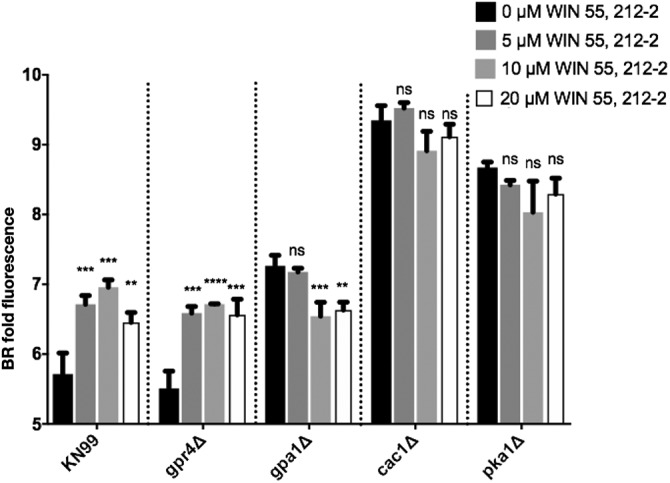
WIN 55,212-2 regulates chitosan through the cAMP/PKA pathway. C. neoformans strains deficient in genes from the cAMP/PKA pathway were cultured with and without WIN 55,212-2 for 3 days, stained with BR, and measured for gMFI by flow cytometry. Means among groups (*n* = 3) were compared to those of strains with no drug added using a one-way ANOVA followed by the Bonferroni multiple-comparison test.

Finally, the role of cAMP in regulating chitosan can also be assessed by the addition of a potential agonist, which should ultimately cause a decrease in cAMP and an associated increase in chitosan. From the primary screen of the ICCB library, we found an increase in BR fluorescence associated with WIN 55,212-2, a known agonist of the cannabinoid receptors CNR1 and CNR2 that causes decreased cAMP in mammalian cells (32, 33). As predicted, incubation of WIN 55,212-2 compounds with wild-type C. neoformans resulted in decreased intracellular cAMP and increased chitosan as measured by BR fluorescence (Fig. 9). Next, we wanted to ascertain if the decrease in intracellular cAMP caused by WIN 55,212-2 was due to the cAMP/PKA pathway. We found that a dose-response of WIN 55,212-2 with the cAMP/PKA pathway mutant strains resulted in an increase of chitosan in the wild type and *gpr4Δ* strain, a decrease of chitosan from *gpa1Δ* strains, but no effect on the *cac1Δ* or *pka1Δ* strain (Fig. 10). This suggests that WIN 55,212-2, similar to BML-190, works through the cAMP/PKA pathway to regulate chitosan, but it does not use Gpr4 as a receptor to induce its effects. Also, interestingly, it induces an inverse agonist effect when Gpa1 is not expressed, suggesting that the core cAMP-producing proteins can be activated by multiple signals.

### BML-190 has physiochemical properties that could enable clinical success.

The rule of five shows that 90% of orally administered drugs that have gone beyond phase II clinical studies have the following physiochemical properties (35): (i) having a molecular weight of ≤500, (ii) having a water partition coefficient (log*P*), predicted by XlogP3, of ≤5, (iii) being a hydrogen bond donor and acceptor of ≤5 and ≤10, respectively, and (iv) having a rotatable-bond count of ≤10. Furthermore, in consideration that fatality associated with cryptococcosis is primarily found to occur due to dissemination into the central nervous system (CNS), drugs that can gain entrance into this location are ideal (36). Criteria identified for this to happen are drug compounds having a polar surface area (PSA) of <70 Å^2^, which will allow them to pass through the blood-brain barrier. Based upon these physical criteria, BML-190 could be a potential clinically successful drug (Fig. S6A); however, its potency is not high. Finally, cytotoxicity (CC_50_) of BML-190 to human hepatoma cells measured by a 3-(4,5-dimethylthiazol-2-yl)-5-(3-carboxymethoxyphenyl)-2-(4-sulfophenyl)-2H-tetrazolium (MTS) assay was not found within the range tested (0.2 μM to 100 μM), suggesting minimal toxicity to humans (Fig. S6B). However, BML-190 also binds to the cannabinoid receptors, suggesting that it would not be without side effects. Together these results indicate that it would have to go through much more optimization before being pursued as a potential drug.

10.1128/mBio.02264-19.6 FIG S6 BML-190 has physiochemical properties that predict its clinical success. (A) Physiochemical properties to help identify the clinical success of an orally administered drug showing the predicted success of BML-190. (B) Cytotoxicity (CC_50_) curve showing minimal toxicity of BML-190 to hepatoma cells, measured by a 3-(4,5-dimethylthiazol-2-yl)-5-(3-carboxymethoxyphenyl)-2-(4-sulfophenyl)-2H-tetrazolium (MTS) assay, that were incubated for 3 days under a compound exposure range of 0.19 μM to 100 μM. Download FIG S6, JPG file, 0.1 MB. Copyright © 2019 Maybruck et al. 2019 Maybruck et al. This content is distributed under the terms of the Creative Commons Attribution 4.0 International license.

**FIG 11:**
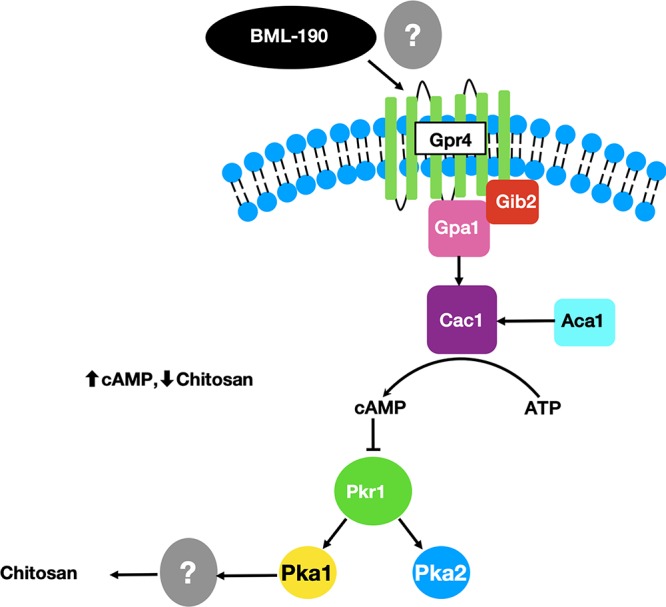
A proposed model for the target and the mode of action of BML-190 in C. neoformans. cAMP from the cAMP/PKA pathway is important for chitosan regulation. The pathway can be induced by BML-190 acting through the G-protein-coupled receptor Gpr4. By analogy of BML-190 in the mammalian system, it is likely that it binds to Gpr4. The induction by BML-190 within this pathway also requires Gpa1, Cac1, and Pka1. Not shown are the potential interactions and cross talk with other signaling pathways, such as the cell integrity/PKC1/MPK1 pathway (44). Pathway illustration adapted from the work of Kronstad et al. (28).

Overall, our results show that cAMP from the cAMP/PKA pathway is important for chitosan regulation. The pathway can be induced by BML-190 acting through the G-protein-coupled receptor Gpr4 (Fig. 11). The induction by BML-190 within this pathway also requires Gpa1, Cac1, and Pka1.

## DISCUSSION

C. neoformans causes life-threatening meningoencephalitis in persons with AIDS. Even with antifungal therapy, mortality hovers around 30%. The current array of antifungals is insufficient to reduce worldwide fungal disease due to inadequate efficacy caused by inherent toxicities and/or emerging drug resistance. These limitations are, in part, due to a paucity of antifungal targets. Therefore, strategies that involve the identification of novel targets that could lead to therapies with increased efficacy are needed. One approach to fulfill this goal is the targeting of factors required for growth in the host, such as chitosan production in C. neoformans. There is some evidence to suggest that drugs targeting virulence factors may reduce the likelihood of the development of antimicrobial resistance. The argument has been made that in the context of natural selection, if a drug does not directly kill the microbe, such as the case when targeting virulence factors, selective pressures are reduced and that the likelihood of the drug selecting for tolerant variants of the target pathogen, and promoting antimicrobial resistance in a population, may also be reduced (37–39). Furthermore, because chitosan deficiency inhibits C. neoformans survival in mammalian hosts (8, 11), even in immunocompromised hosts (data not shown), and chitosan is not produced by mammals, chitosan biosynthesis may be an ideal novel drug target for the treatment of cryptococcosis, in particular, in the context of AIDS patients who are CD4 T cell deficient.

Through our development of a novel and robust flow cytometry primary screen for compounds that reduce chitosan production, we identified a compound, BML-190, that reduces chitosan. BML-190 is an inverse agonist targeting the G-protein-coupled human cannabinoid receptors CNR1 and CNR2. It induces adenylyl cyclase activity and causes an intracellular accumulation of cAMP (24). Since G-protein signaling is known to be conserved among organisms, and the cAMP/PKA signaling pathway that regulates cAMP is known within C. neoformans, we explored the importance of a receptor for this pathway, Gpr4, as a target for BML-190. We treated known GPCR gene deletions, *gpr3Δ* (Fig. S4), *gpr2Δ*, *gpr4Δ*, and *gpr5Δ*, with BML-190 and showed that in all but the *gpr4Δ* strain, BML-190 was able to cause a reduction of chitosan (Fig. 4A).

We found increased cAMP levels in wild-type cells treated with BML-190 but not in *gpr4Δ* cells (Fig. 4B). We further tested our hypothesis that cAMP levels governed by the cAMP/PKA pathway regulate chitosan levels by the use of another small molecule identified in our screen. We showed a decrease in cAMP and an increase in chitosan in cells when we treated cells with WIN 55,212-2, an agonist toward CNR1 and CNR2 (33), supporting the involvement of the cAMP/PKA pathway (Fig. 8). Furthermore, another confirmed hit from our screen, dipyridamole, is a known phosphodiesterase inhibitor that facilitates the intracellular accumulation of cAMP by preventing its hydrolysis into inactive AMP (40, 41). This compound also shows a dose-dependent reduction in chitosan (Fig. S3). Our work has not directly shown that BML-190 or WIN 55,212-2 binds to Gpr4, or the specific mechanism of action of these two compounds, and further experimentation is needed to conclusively determine binding. However, by analogy to the mammalian system and the binding of these two compounds to CNR1 and CNR2, along with our data showing nonresponse to these compounds in a *gpr4Δ* strain, Occam’s razor (the law of parsimony) would predict that Gpr4 is their target.

C. neoformans PKA is a tetrameric protein that is made up of a dimeric regulatory subunit (Pkr1) and two monomeric catalytic subunits (Pka1/2). Upon cAMP targeting of Pkr1, PKA becomes activated and releases Pka1/2, which activates downstream targets by phosphorylation. It has been noted that most of the physiological effects from cAMP in fungi are mediated through targeting PKA (42, 43). To provide intracellular cAMP for PKA targeting, Gpr4 acts as a receptor for glucose and methionine to trigger signaling through the cAMP/PKA pathway (29). When Gpr4 is physically associated with Gpa1, the adenylyl cyclase enzyme (Cac1) is activated to convert ATP into cAMP.

One important effect of PKA, through this pathway, is that it has been noted to regulate C. neoformans virulence. Specifically, *pka1Δ*, *gpa1Δ*, and *cac1Δ* mutant strains have reduced virulence along with reduced melanin and capsule volume (28, 43, 44). Furthermore, PKA is known to promote cell proliferation and negatively regulates cAMP, acting as a negative-feedback loop for PKA activity via its activation of phosphodiesterase (Pde1 [34]). Since we showed that chitosan production is inversely related to cAMP levels (Fig. 2C and D and Fig. 8) and that there is no decrease in chitosan in the *pka1Δ*, *gpa1Δ*, and *cac1Δ* mutant strains with increasing concentrations of BML-190 (Fig. 5), it can be further supported that the mode of action of BML-190 is the cAMP/PKA pathway. Interestingly, the chitosan levels are higher in these mutant strains than in the wild type with/without BML-190. Since we have noted that there is an inverse relationship between chitosan and cAMP production, lower cAMP should produce greater chitosan. This would be the case in *gpa1Δ* and *cac1Δ* mutants since the corresponding proteins are noted to be important for cAMP formation. However, this does not explain the higher chitosan production in *pka1Δ* mutants, which should have relatively large amounts of cAMP (34) because Pde1 is not activated by Pka1 to degrade cAMP. One possibility is that cAMP is negatively regulated by mechanisms in addition to Pde1. Also, through our dose-response of adding exogenous cAMP to *pka1Δ*, in turn, causing a loss of chitosan (Fig. 8), it suggests that cAMP levels are indeed low in the examined cAMP/PKA deletion strains, which conforms to our hypothesis of the inverse relationship between cAMP and chitosan. It is possible that *pka1Δ* mutants grown under optimal growth conditions (i.e., YPD growth conditions) show greater cAMP levels than those grown under host conditions (i.e., cRPMI growth at 37°C and 5% CO_2_), the latter of which were used for this study.

As previously stated, PKA is a well-described mediator of the effects of cAMP on inducing virulence factors in C. neoformans. This has been identified to include the organism’s ability to proliferate under host conditions (37°C) and to produce an antiphagocytic polysaccharide capsule and antioxidant melanin (28, 43) and now chitosan as well. Surprisingly, not only does BML-190 reduce chitosan, but also our data suggest that it reduces capsule volume and melanin (Fig. S7 and S8). Based upon our evidence that BML-190’s mode of action is the cAMP/PKA pathway, with cAMP targeting PKA to induce a mechanism downstream to cause a reduction in chitosan, it is also possible that this downstream mechanism is responsible for causing a reduction in the other virulence factors of capsule volume and melanin as well as decreased growth at 37°C (Fig. 2). Future studies will also examine the effects of BML-190 on Crg2 since it has been identified to be important in the cAMP/PKA pathway in negatively regulating cAMP, in turn facilitating C. neoformans virulence (45). Another possibility is that cross talk occurs between another pathway involved in regulating virulence factors such as the cell wall integrity (CWI) pathway, which we have shown interacts with the cAMP/PKA pathway (46). However, this mechanism has yet to be elucidated.

10.1128/mBio.02264-19.7 FIG S7 BML-190 causes a decrease in capsule volume. Wild-type C. neoformans (KN99α) was grown in cRPMI (0.625% HI-FBS) and 0.1% DMSO with and without 20 μM BML-190 for 3 days at 37°C and 5% CO_2_. Cells were then washed in 1× PBS and resuspended in 1/4 dilution of India ink (50 μl) and then added (10 μl) to a microscope slide. The diameters of the whole cell and cell body were measured from 60 different cells within each biological replicate. Volume of a sphere was determined from those diameters. Capsule volume was determined by subtracting the volume of the cell body from the volume of the whole cell. Means between groups (*n* = 3) were compared using a Student *t* test. **, *P*< 0.01. Download FIG S7, PDF file, 0.8 MB. Copyright © 2019 Maybruck et al. 2019 Maybruck et al. This content is distributed under the terms of the Creative Commons Attribution 4.0 International license.

10.1128/mBio.02264-19.8 FIG S8 BML-190 causes a decrease in C. neoformans melanin. Wild-type C. neoformans (KN99α) was grown in cRPMI (0.625% HI-FBS) and 0.1% DMSO with and without 20 μM BML-190 for 3 days at 37°C and 5% CO_2_ (*n* = 3). Cells were then washed in 1× PBS. A total of 1 × 10^8^ cells from each condition described above were added to 2 ml of glucose-free asparagine medium for 7 days at 30°C. Download FIG S8, JPG file, 0.1 MB. Copyright © 2019 Maybruck et al. 2019 Maybruck et al. This content is distributed under the terms of the Creative Commons Attribution 4.0 International license.

In conclusion, the paucity of drug targets and the lower efficacy of current treatments for cryptococcosis, a prominent cause of morbidity and mortality in AIDS patients, mean that additional treatments must be discovered. To help increase efficacy, drugs and their targets must be found that target the pathogen and are nontoxic to the host. By targeting chitosan of the pathogen, we provide a strategy that fulfills those criteria. However, because BML-190 also has been shown to have effects on cannabinoid receptors, it is unlikely to be developed further as an anticryptococcus drug, but additional screening for reduced chitosan using the flow cytometry cell-based screen could identify compounds that do not impact the mammalian host. Furthermore, by the drug disarming the pathogen, as opposed to killing it within the host, it is possible that there may be a reduced effect of natural selection by the drug on the pathogen and, in turn, a potential reduction in their antimicrobial resistance to the drug, but this remains to be determined.

Our discovery that BML-190 negatively regulates chitosan via the cAMP/PKA signaling pathway also suggests that our screening strategy can identify more compounds from additional drug libraries that will regulate chitosan production from C. neoformans to further identify key genes and proteins critical for the production and regulation of chitosan and cell wall biosynthesis.

## MATERIALS AND METHODS

### *Cryptococcus* strains and growth conditions.

C. neoformans strain KN99α, the mCherry reporter strain (KN99mCH [47]), and strains engineered that differentially express chitosan, i.e., *cda1*Δ, *cda2*Δ *cda3*Δ, and *cda1*Δ *cda2*Δ *cda3*Δ strains (the last two referred to as *cda2Δ3Δ* and *cda1Δ2Δ3Δ* strains, respectively, throughout this report), have been described previously (9). Additional strains used included those derived from KN99α with single deletions in G-protein-coupled receptors, G-protein alpha subunits, and the cAMP/PKA pathway that were part of the deletion library generated by Hiten Madhani at the University of California, San Francisco (UCSF), and obtained from the Fungal Genetic Stock Center (FGSC [30, 31]). These deletion strains (followed by gene identifier [ID]) were *gpr2*Δ (CNAG_01855), *gpr3Δ* (CNAG_03846), *gpr4Δ* (CNAG_04730), *gpr5Δ* (CNAG_05586), *gpa1Δ* (CNAG_04505), *gpa2Δ* (CNAG_00179), *gpa3Δ* (CNAG_02090), *cac1Δ* (CNAG_03202), and *pka1Δ* (CNAG_00396) strains. All strains were streaked onto yeast-peptone-dextrose (YPD) agar plates from –80°C stocks. After the development of colonies, strains were further propagated by their addition to YPD broth and grown for 72 h at 30°C and 300 rpm.

### Flow cytometry cell-based phenotypic screening assay.

We screened 480 compounds from the Institute of Chemistry and Cell Biology (ICCB) Known Bioactives library (Enzo Life Sciences) using our optimized cell-based flow cytometry phenotypic assay. We used a Biomek FX workstation to add 1 μl of each compound to columns 2 to 11 of 96-well sterile round-bottom tissue culture plates in duplicate. For the controls, 1 μl of DMSO was added to columns 1 and 12. The screening strain (*cda2Δ3Δ*) and the chitosan-deficient strain (*cda1Δ2Δ3Δ*) were initially cultured in the complete growth medium YPD, washed twice in 1× PBS, counted by hemocytometer, and resuspended at 5 × 10^5^ cells/ml in complete RPMI (cRPMI; 0.625% HI-FBS). We added 100 μl of cells per well, for a final DMSO concentration of 1%, which ensured the solubility of the ICCB compounds. We found minimal impact on growth of either strain under these conditions (data not shown). Plates were incubated for 3 days at 37°C with 5% CO_2_. After incubation, we washed the cells with 1× PBS and stained them with Cibacron brilliant red 3B-A (BR) for 20 min at a final concentration of 5 μg/ml in PBS, pH 2. We resuspended one well of the screening strain in 1× PBS at pH 2 without BR as an unstained control to measure background fluorescence. Cells were then pelleted via centrifugation at 1,000 × *g* for 10 min at room temperature in the plates, flick decanted, and fixed by resuspension in 1% paraformaldehyde (PFA) followed by overnight incubation at 4°C. Prior to flow cytometry we centrifuged the plates and flick decanted and resuspended the cells in flow cytometry buffer (0.5% bovine serum albumin [BSA] plus 2 mM EDTA plus 1× PBS). All plates were then analyzed for multiple parameters using a BD Biosciences LSRFortessa X-20 flow cytometer equipped with a high-throughput sampler (HTS), which enables automated sample acquisition from 96- and 384-well microtiter plates. BR fluorescence was detected by excitation with a yellow-green laser (561 nm) and emission collection with the PE channel (586/15 nm). We were able to complete the analysis of 12 plates (1,152 wells = compounds + controls) in 6.5 h. The average Z-prime for these 12 plates was 0.51, indicative of a robust screening assay (14).

### 3-Methyl-2-benzothiazolone hydrazone hydrochloride (MBTH) chitosan assay.

C. neoformans strains were grown in 25 ml of cRPMI (0.625% HI-FBS) at 5 × 10^5^ cells/ml under tissue culture conditions for 72 h at 37°C and 5% CO_2_. Cells were then pelleted into preweighed 15-ml conical centrifuge tubes by centrifugation (3,000 × *g* for 10 min). The supernatant was aspirated and then samples were resuspended with 1× PBS and the cells were again pelleted. Pelleted cells were then weighed after being freeze-dried to normalize chitosan measurements to dry weights. To reduce background chromogens, cells were alkaline extracted by the addition of 10 ml of 6% KOH to cell pellets, followed by incubation at 80°C for 90 min and periodic mixing. Pellets were then washed twice with 10 ml of 1× PBS followed by two washes in ultrapure water. Samples were then all normalized to their initial dry weights by addition of ultrapure water to make all samples 10 mg/ml.

For MBTH colorimetric determination of chitosan, samples of 100 μl (1 mg) were diluted 1:1 with 1 M HCl. Then 400 μl of 0.36 M sodium nitrite was added, vortexed, and incubated for 15 min at room temperature. Next 200 μl of 1.1 M ammonium sulfamate was added, vortexed, and incubated for 5 min at room temperature. This was then followed by the addition of 200 μl of 0.011 M MBTH, followed by vortexing and incubation for 30 min at 37°C. To induce blue color formation, 200 μl of 0.018 M iron(III) chloride hexahydrate was added and vortexed, followed by incubation for 5 min at 37°C. Samples were cooled to room temperature and centrifuged at 14,000 rpm for 2 min. One hundred microliters of supernatant from each sample was added to wells of a flat-bottom 96-well microtiter plate and absorbance was measured at 650 nm. A standard curve was prepared from 2-fold serial dilutions of 2 mM GlcN for assessment of chitosan concentrations of samples.

### cAMP measurements.

We grew C. neoformans strains overnight in YPD, washed the cells twice in 1× PBS, and added them at a concentration of 5 × 10^8^ cells/ml to T-175 tissue culture flasks containing 10 ml of cRPMI (0.625% HI-FBS) with a final concentration of 1% DMSO with or without the testing compound. We incubated the flasks for 30 min at 37°C and 5% CO_2_ and then collected medium from each flask and centrifuged it at 3,000 × *g* for 10 min at 4°C. We aspirated the media and resuspended the cell pellets in 2 ml of ice-cold 0.1 M HCl, transferring 1.5 ml to 2-ml screw-top microtubes (Sarstedt Inc., Numbrecht, Germany) on ice that contained 0.5-mm zirconia/silica beads (Biospec Products, Bartlesville, OK). Cells were homogenized using a Mini-Beadbeater-16 (Biospec Products) at 75% of maximum agitation at 4°C for 3 rounds of 2 min on and 2 min off. We transferred the homogenized samples to new tubes and stored them at –20°C. We thawed the samples on ice, centrifuged them at 3,000 × *g* for 10 min, and transferred the supernatants to fresh tubes. We measured cAMP concentrations using a cAMP competitive enzyme-linked immunosorbent assay (ELISA) kit according to the manufacturer’s instructions (Sigma-Aldrich, St. Louis, MO). Samples were added to ELISA wells as 100-μl aliquots (2.5 × 10^8^ cell lysate equivalent) and were not acetylated prior to measurements.

### BR staining of C. neoformans for fluorescence microscopy.

We dissolved 10 mg/ml of BR in ultrapure water. *Cryptococcus* cells grown in YPD were washed once in PBS (1,000 × *g* for 10 min) and diluted to 1 × 10^7^/ml of PBS. Cells were then pelleted and resuspended in 5 μg/ml of BR in PBS (pH 2), followed by incubation at room temperature for 20 min in the dark. We washed the cells with 1× PBS and resuspended them at 4 × 10^7^/ml in PBS. We transferred 10 μl to a microscope slide, covered it with a coverslip, and sealed with nail polish. Slides were then examined by epifluorescence using filter sets for tetramethyl rhodamine isocyanate (TRITC) (excitation, 521 to 565 nm; emission, 553 to 633 nm).

### Capsule measurements.

Wild-type C. neoformans (KN99α) cells were grown in cRPMI (0.625% HI-FBS) and 0.1% DMSO with or without 20 μM BML-190 for 3 days at 37°C and 5% CO_2_. Cells were then washed in 1× PBS and resuspended in 1/4 dilution of India ink (50 μl) and then added (10 μl) to a microscope slide. The diameters of the whole cell and cell body were measured from 60 different cells within each biological replicate. Volume of a sphere was determined from those diameters. Capsule volume was determined by subtracting the volume of the cell body from the volume of the whole cell. Means between groups (*n* = 3) were compared using a Student *t* test.

### Melanin measurements.

Wild-type C. neoformans (KN99α) was grown in cRPMI (0.625% HI-FBS) and 0.1% DMSO with or without 20 μM BML-190 for 3 days at 37°C and 5% CO_2_ (*n* = 3). Cells were then washed in 1× PBS. Then 1 × 10^8^ cells (5 × 10^7^/ml), from each condition described above, were added to 2 ml of glucose-free asparagine medium (1 g/liter of l-asparagine, 0.5 g/liter of MgSO_4_·7 H_2_O, 3 g/liter of KH_2_PO_4_, and 1 mg/liter of thiamine, plus 1 mM l-3,4-dihydroxyphenylalanine [l-DOPA]) for 7 days at 300 rpm and 30°C. Samples were then spun down at >600 × *g* for 10 min. Pellets were then transferred to a 96-well flat-bottom microtiter plate and photographed.

### Statistical analysis.

All data from experiments are from greater than or equal to three independent experiments. Error bars represented standard deviations. Inferential statistics used at least one of the following relevant parametric analyses: a two-tailed unpaired Student *t* test, a one-way analysis of variance (ANOVA) with Bonferroni multiple-comparison test, and/or a Pearson’s *r* correlation analysis. For all experiments, statistically significant differences are indicated in figures as follows: *, *P ≤ *0.05; **, *P ≤ *0.01; ***, *P ≤ *0.001; and ****, *P ≤ *0.0001. All statistical analysis was completed on Prism 7 for Mac OS X software (version 7.0c).

### Data availability.

The screening data have been deposited in the publicly available PubChem database under identifying number AID 1347148 (https://pubchem.ncbi.nlm.nih.gov/bioassay/1347148).
