# Supplementary figures and images for: The Aminoalkylindole BML-190 Negatively Regulates Chitosan Synthesis via the Cyclic AMP/Protein Kinase A1 Pathway in Cryptococcus neoformans
Source: mBio. 2019 Dec 17;10(6):e02264-19. doi: 10.1128/mBio.02264-19 (PMC6918072; doi:10.1128/mBio.02264-19)

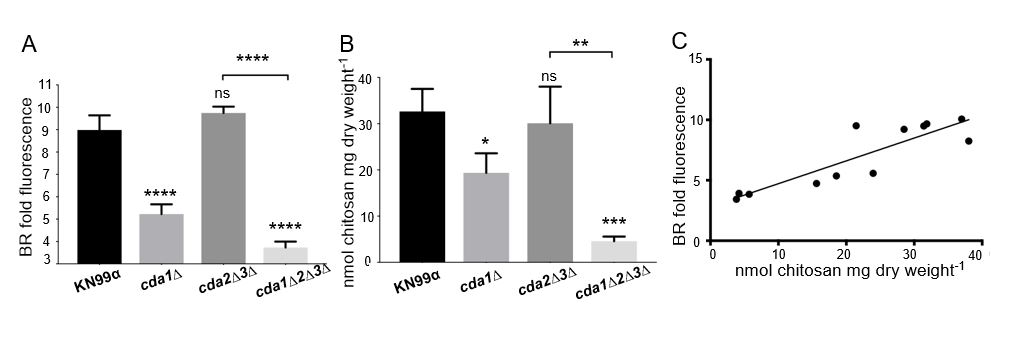

Supplement: FIG S1 [file mBio.02264-19-sf001.jpg]

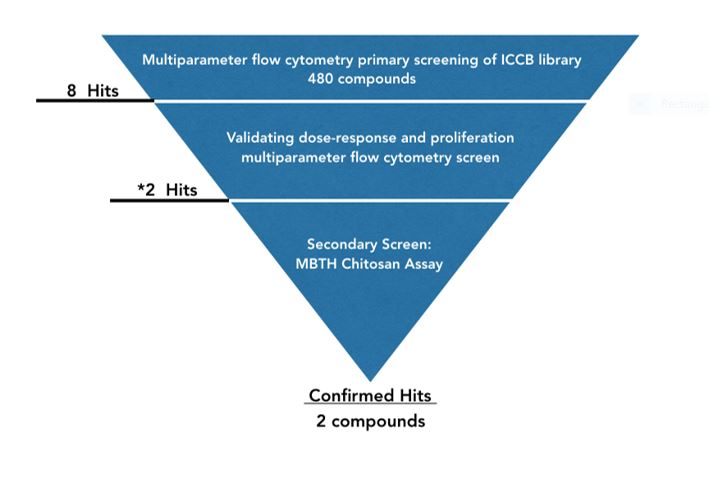

Supplement: FIG S2 [file mBio.02264-19-sf002.jpg]

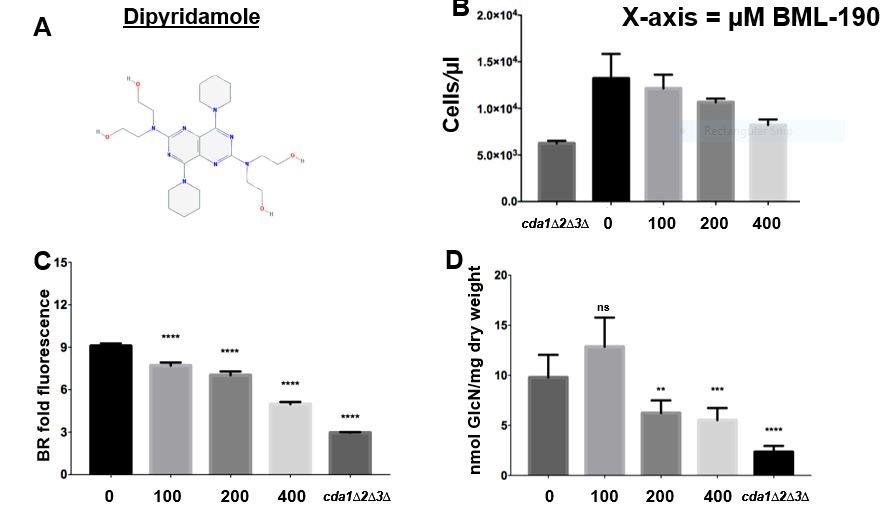

Supplement: FIG S3 [file mBio.02264-19-sf003.jpg]

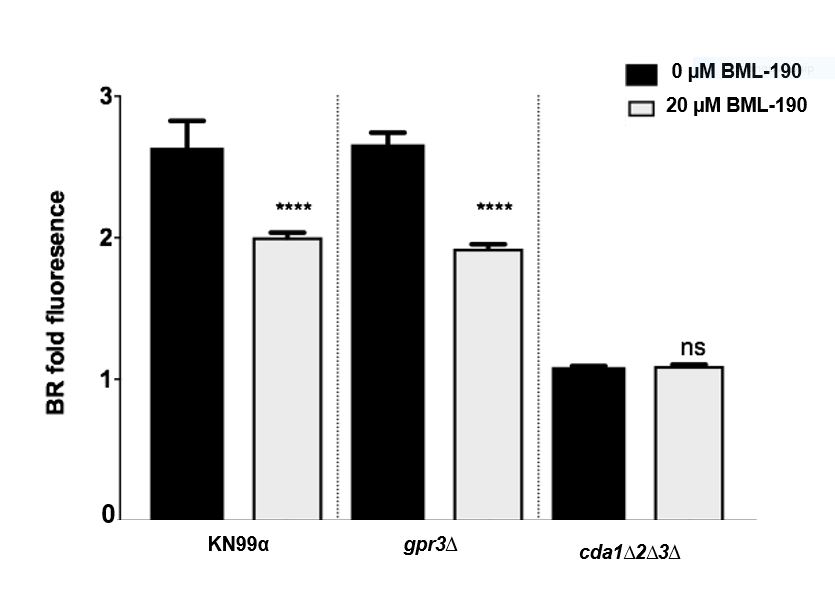

Supplement: FIG S4 [file mBio.02264-19-sf004.jpg]

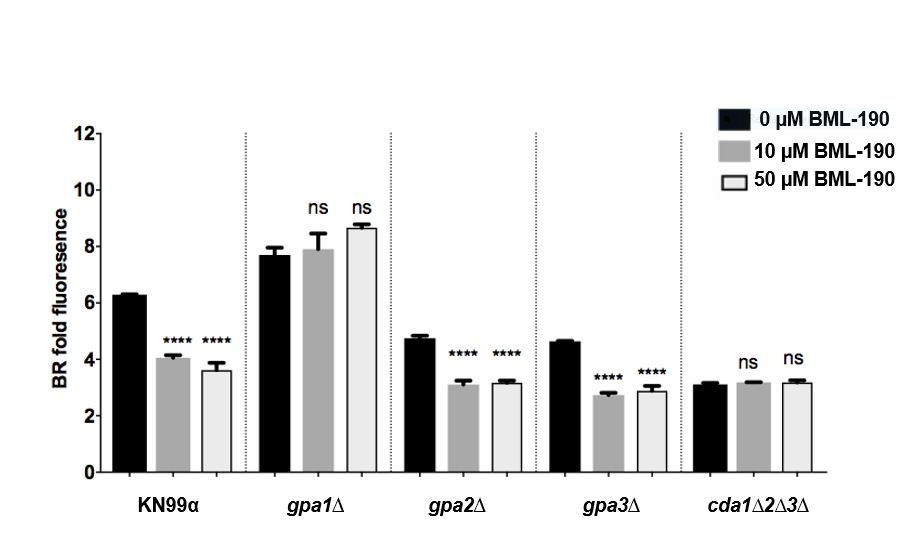

Supplement: FIG S5 [file mBio.02264-19-sf005.jpg]

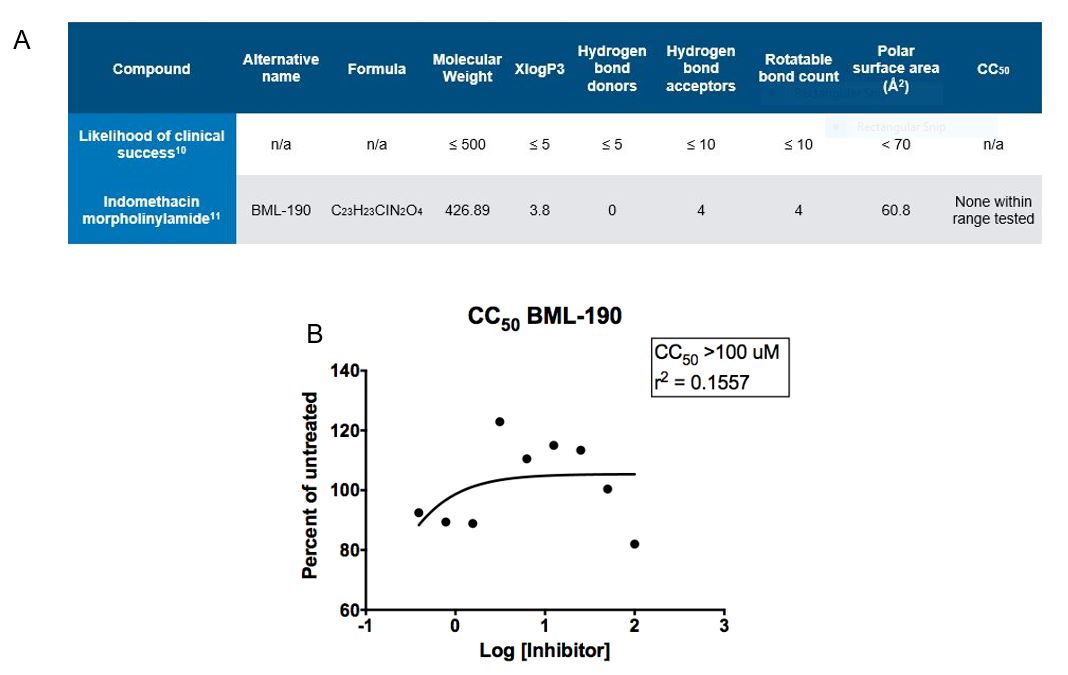

Supplement: FIG S6 [file mBio.02264-19-sf006.jpg]

**0  $\mu$ M BML-190**

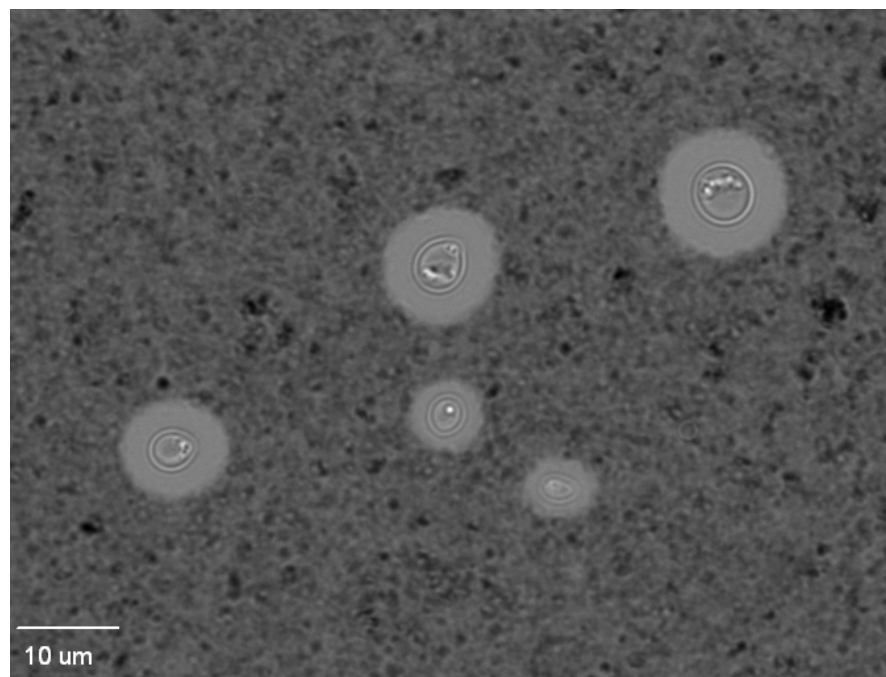

**20  $\mu$ M BML-190**

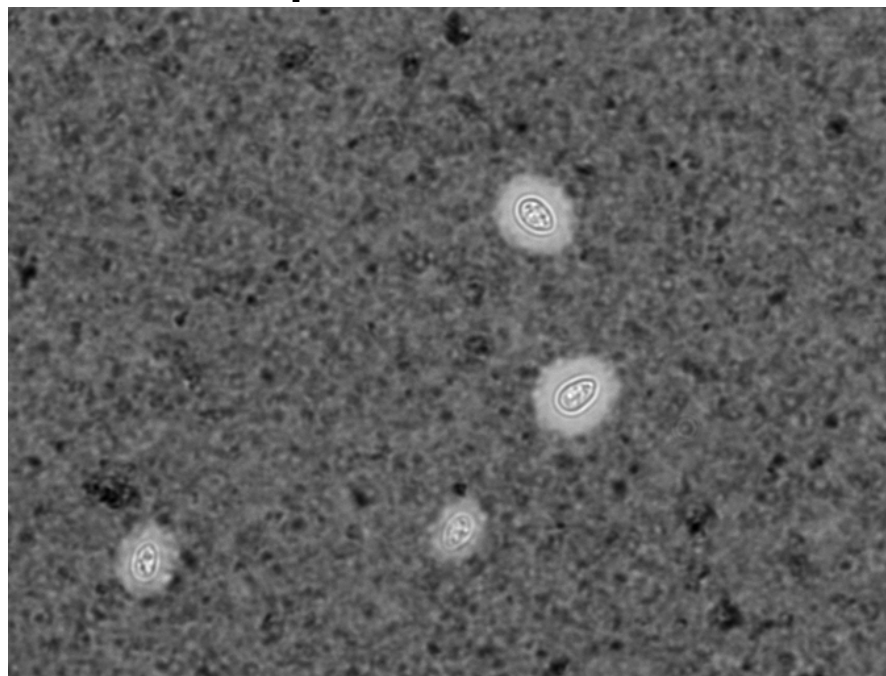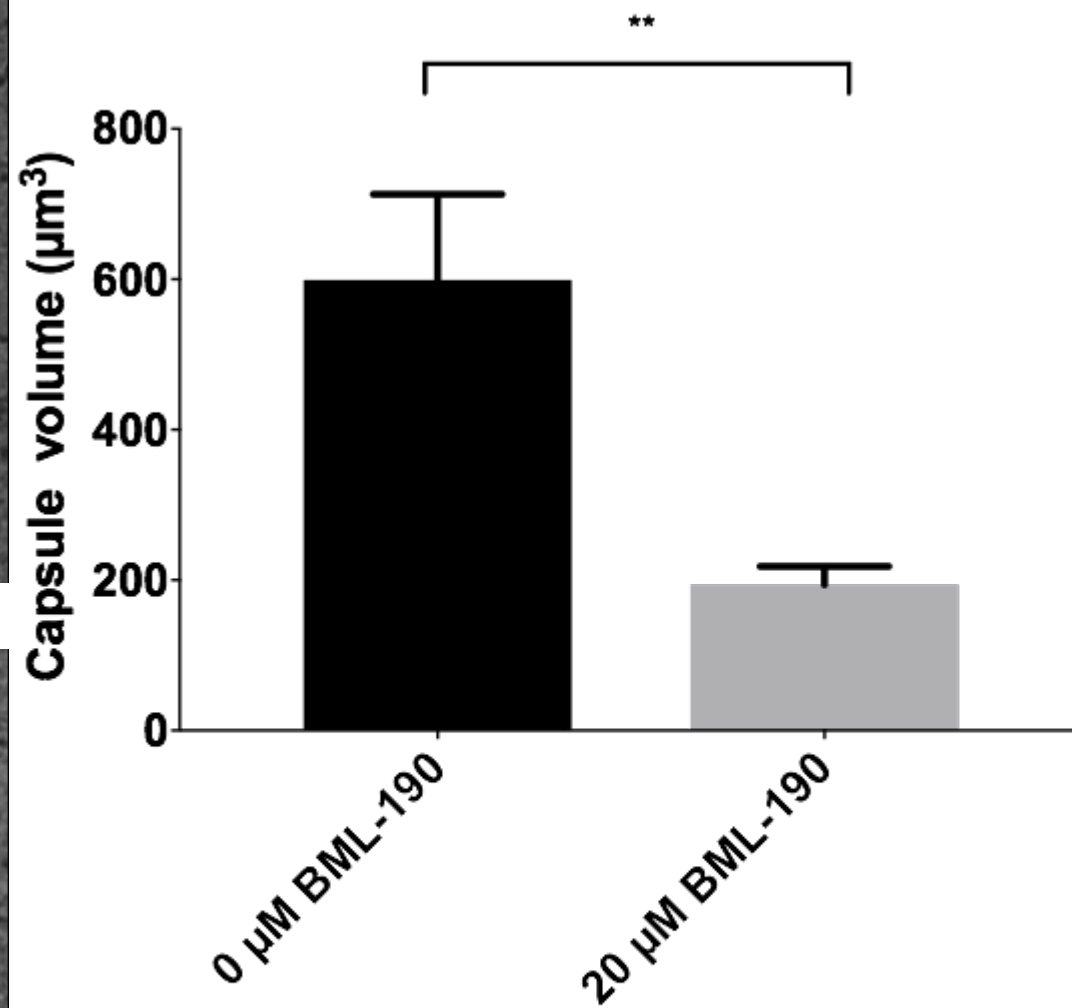

Supplement: FIG S7 [file mBio.02264-19-sf007.pdf]

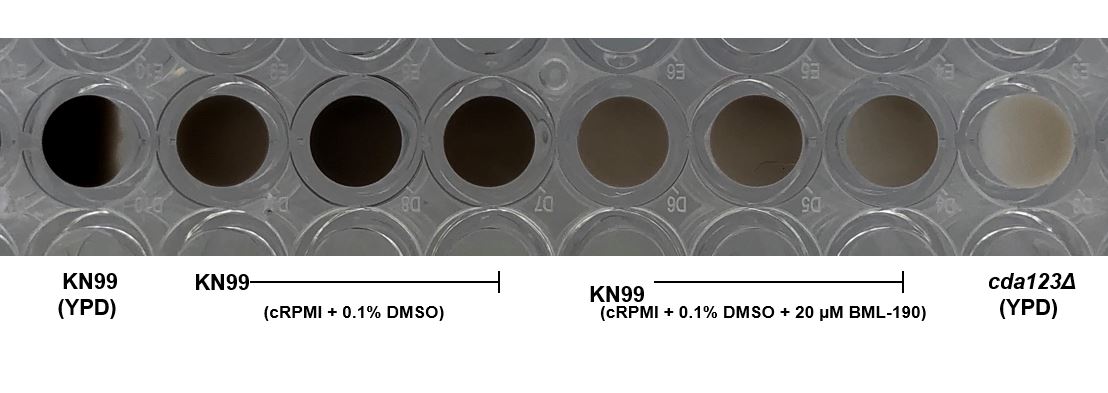

Supplement: FIG S8 [file mBio.02264-19-sf008.jpg]
